# Simultaneously Acquired Magnetoencephalography and Diffuse Optical Tomography Data Reveals Correlated Somatosensory Activity

**DOI:** 10.1002/hbm.70293

**Published:** 2025-07-27

**Authors:** Salla Autti, Pauliina Hirvi, Mariia Keitaanniemi, Hanna Mustaniemi, Kalle Kotilahti, Hanna Renvall, Ilkka Nissilä

**Affiliations:** ^1^ Department of Neuroscience and Biomedical Engineering Aalto University Espoo Finland; ^2^ BioMag Laboratory, HUS Medical Imaging Center Aalto University, University of Helsinki and Helsinki University Hospital Helsinki Finland; ^3^ Department of Mathematics and Systems Analysis Aalto University Espoo Finland

**Keywords:** diffuse optical tomography, functional near‐infrared spectroscopy, magnetoencephalography, median nerve, multimodal neuroimaging, somatosensory cortex

## Abstract

Simultaneous measurement of electrophysiological and hemodynamic brain signals imposes special requirements on the instrumentation. Here, we developed a high‐density fiberoptic probe for concurrent diffuse optical tomography (DOT) and magnetoencephalography (MEG) recordings. Transparent two‐component silicone was mixed with carbon black dye to achieve a black, flexible, non‐magnetic support for the dense optode arrangement and low (5 mm) probe thickness. The probe was used to record somatosensory responses to electrical right median nerve stimulation at 0.5, 1, 2, and 4 Hz in 18 adult human subjects. Brain activity was simultaneously measured with a commercial whole‐head MEG system and with the DOT optode arrangement covering approximately 40 cm^2^ over the parietal region in the contralateral left hemisphere. Two correlation‐based clustering methods were developed to find regions where the reconstructed time course of total hemoglobin concentration (HbT) changes correlated with the predicted hemodynamic activity based on time‐course characteristics of the MEG sources and the canonical hemodynamic response model. Two statistically significant clusters were found based on the correlation between HbT around the postcentral gyrus and MEG primary somatosensory cortical activity at ~35 ms (P35m response). In addition, correlation between HbT and secondary somatosensory cortical activity suggested a statistically significant cluster in the postcentral gyrus and parietal operculum. These results illustrate an improvement in localization over previous DOT studies using sparse optode arrangements, and demonstrate the feasibility of the system for simultaneous HD‐DOT‐MEG experiments. Furthermore, the techniques described here pave the way for understanding the coupling between hemodynamic and electrophysiological responses. Further research is needed to reveal the neuronal circuits giving rise to the correlating MEG and DOT response features. Significant improvements in the technology are still expected via optimization of the detected light power in the instrumentation.

## Graphical abstract

Fiberoptic probe for simultaneous high‐density diffuse optical tomography (HD‐DOT) and magnetoencephalography (MEG) was constructed and tested in a somatosensory experiment. Equivalent current dipoles were fitted to the MEG data. SI and SII dipole waveform features were extracted and used to localize correlated hemodynamic activity in the postcentral gyrus.

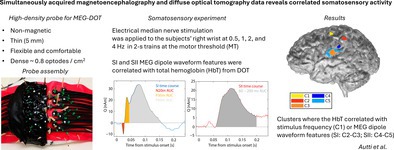

**Summary**

- Keypoints
  ○ Non‐magnetic, high‐density diffuse optical tomography (HD‐DOT) probe was constructed.
  ○ DOT and magnetoencephalography (MEG) data were acquired simultaneously during right median nerve stimulation at 0.5, 1, 2, and 4 Hz.
  ○ Evoked magnetic fields correlated with the total hemoglobin concentration in the left postcentral gyrus.
- Practitioner's points
  ○ HD‐DOT and MEG data can be simultaneously acquired to investigate electrophysiological and hemodynamic brain signals.
  ○ Total hemoglobin concentration acquired with high‐density optode arrangement provides good spatial localization.

## 1 Introduction

The macroscopic electrical and magnetic fields created by electrical activity of large populations of neurons can be recorded non‐invasively with electro‐ and magnetoencephalography (EEG and MEG, respectively). While these electrophysiological signals provide a direct measure of neuronal activity, synaptic activity also regulates vascular diameter, cerebral blood flow (CBF), and volume (CBV) in nearby arterioles through neurovascular coupling. This ensures adequate blood supply to neurons and supporting glial cells during and after neuronal activation (Iadecola 2017). Neuronal activity also consumes oxygen and glucose supplied by the bloodstream. These vascular and metabolic changes lead to hemodynamic signals that provide an indirect view of neuronal activity. Changes in the concentrations of oxygenated and deoxygenated hemoglobin (HbO_2_ and HbR, respectively) cause inhomogeneities in the magnetic field which can be detected via the blood‐oxygen‐level‐dependent (BOLD) signal in functional magnetic resonance imaging (fMRI; (Ogawa et al. 1998)). HbO_2_ and HbR are significant absorbers of near‐infrared light, and changes in their concentrations can be measured using diffuse optical imaging (DOI) techniques including functional near‐infrared spectroscopy (fNIRS; (Villringer et al. 1993)) and diffuse optical tomography (DOT) which uses a light propagation model and image reconstruction algorithm to make 3D images of optical absorption and scattering (Arridge 1999).

The electrophysiological and hemodynamic measures may provide important complementary information on the underlying brain functions. So far, most studies on the relationship between electrophysiological and hemodynamic signals have been carried out in animals (see, e.g., Logothetis et al. (2001); Devor et al. (2003)). Ideally, such measures should be acquired simultaneously, but this imposes special requirements on the instrumentation, especially for human studies. In the present study, we used simultaneous MEG and DOT measurements in human subjects during electrical stimulation of the median nerve. Somatosensory cortical responses are particularly well characterized in the previous literature; see, for example, Hari and Forss (1999), facilitating direct comparisons to earlier results. The earliest deflection in the somatosensory evoked potentials (SEPs in EEG) and somatosensory evoked fields (SEFs in MEG), N20 (N20m in MEG), is known to reflect excitatory postsynaptic potentials in the Brodmann cytoarchitectonic area 3b in the primary somatosensory cortex (SI). It is followed by deflections typically at around 35 ms (P35) and 60 ms (P60) also in the SI. The bilateral secondary somatosensory cortices (SIIs) in the Sylvian fissures are active around 60–120 ms after the onset of the median nerve stimulus (Mauguiere et al. 1997; Raij et al. 2003): The SII has been suggested to play a role, for example, in somatosensory recognition (Dijkerman and De Haan 2007). In repeated stimulation, the refractory behavior of SI and SII responses greatly depends on the interstimulus interval (ISI) (Wikström et al. 1996; Raij et al. 2003). In addition to the SI and SII areas, peripheral nerve stimulation has been shown to activate the primary motor cortex (M1), premotor cortex, supplementary motor area, posterior insula, rostral parts of the posterior parietal cortex (PPC), and the parietal operculum in stereo‐EEG measurements (Avanzini et al. 2018).

Hemodynamic responses to median nerve stimulation have been investigated using fNIRS and fMRI. fNIRS studies have reported the activation of SI (Franceschini et al. 2003; Tanosaki et al. 2003), while in BOLD fMRI, the SI (Spiegel et al. 1999), bilateral SII (Nihashi et al. 2005), frontal cortex (Chen et al. 2008), and ipsilateral cerebellum (Backes et al. 2000) have been shown to activate. In contrast to the refractory behavior observed with electrophysiological recordings, HbO_2_ responses to median nerve stimulation increased at higher stimulation frequencies up to 20 Hz in adult humans (Tanosaki et al. 2003; Ferretti et al. 2007; Takeuchi et al. 2009).

In addition to providing a more comprehensive view of brain function, multimodal imaging may be used to overcome limitations of individual methods and help to understand how the measurements from individual modalities relate to each other. In these efforts, the combination of EEG and NIRS has often been chosen due to their relative portability, low cost, and availability, as well as the widespread clinical and research use of EEG; for a review of simultaneous EEG and NIRS recordings, see Li et al. (2022). Simultaneous EEG and optical recordings of electrical forepaw stimulation in anesthetized rats (Franceschini et al. 2008) revealed the relationship between SEP features and hemoglobin signals, that is, HbO_2_, HbR, and the total hemoglobin concentration (HbT) as a function of stimulus frequency (1–8 Hz presented in 4 s trains) to be nonlinear, with 2 Hz stimulation giving the largest HbO_2_ response area‐under‐the‐curve (AUC) and the 20 ms SEP peak having the closest linear correspondence with the hemodynamic signal. In contrast, in humans, EEG and NIRS signals recorded simultaneously during median nerve stimulation at varying frequencies (2–10 Hz) with long stimulus trains (30 s) (Takeuchi et al. 2009) suggested an increase in mean HbO_2_ response at higher stimulation frequencies. In the first combined direct‐current MEG (DC‐MEG) and NIRS measurement (Mackert et al. 2008), the MEG and time‐resolved NIRS responses closely followed the finger‐movement task cycles. Activation of SI to median nerve stimulation has also been reported in simultaneous MEG and fNIRS and DOT using sparse optode arrangements (Ou et al. 2009; Huppert et al. 2017).

High‐density (HD) optode arrangements for DOT were first shown to be effective in mapping the visual cortex (Zeff et al. 2007). A significant improvement in image quality was achieved by using many partially overlapping source–detector pairs covering the brain area of interest compared to sparse optode arrangements (in children by Heiskala et al. (2009) and Shekhar et al. (2024), and in adults by White and Culver (2010)). In the present study, we constructed a high‐density, non‐magnetic, fiberoptic probe optimized for simultaneous MEG‐DOT recordings, and introduced a novel methodology and implementation for the measurements and data analysis. Both MEG and DOT have a relatively easy subject preparation phase, and measurements can be carried out on a variety of subjects, including young children (Hari et al. 2018; Nevalainen et al. 2012; Medvedovsky et al. 2012; Jönsson et al. 2018; Maria et al. 2022, 2020). The silicone‐supported DOT probe is soft and holds the fibers stable, minimizes artifacts from mechanical contacts between the probe and the MEG dewar, and prevents direct light leaks between optodes.

During the measurements, the right median nerve was electrically stimulated at four different stimulus frequencies (0.5, 1, 2, and 4 Hz) in 18 healthy adult subjects. Equivalent current dipole (ECD) modeling was used for MEG source localization, and atlas model‐based image reconstruction was used to obtain 3D voxel‐based images of the DOT responses. We used a novel data analysis approach where MEG SI and SII dipole time‐course features (N20m, P35m, etc.) were correlated with DOT HbT responses in the gray matter at the voxel‐level, and activation clusters were formed from adjacent statistically significant voxels. The areas with significant correlation between modalities were hypothesized to be either the source of both signals or closely connected to the primary sources of neuronal activity. Finally, we provide interpretations of the obtained results and compare them to previously published multimodal imaging literature.

## 2 Materials and Methods

### 2.1 Study Participants

A total of 22 adult volunteers were recruited for the study. All subjects were right‐handed and without neurological or psychiatric disorders. All subjects gave a prior written informed consent to participate in the study. The ethical statement for the measurement protocol was obtained from the Helsinki University Central Hospital (HUS) regional committee on medical research ethics. After rejecting four subjects due to excessive outside electronic noise during the measurements, missing or low‐quality registration data, or missing MRI, 18 subjects (17 male and one female) of age 21–41 years (mean ± standard deviation [SD] 25 ± 5 years) were included in the final data analysis. In this pilot approach, we focused on short‐haired subjects. With short hair, the DOT array is easier to attach firmly which reduces motion artifacts. Additionally, as the hair is pressed between the skin and the probe, the average attenuation caused by very short hair is lower than with equivalent long hair. In seven subjects, the hair length varied between 0 and 5 cm, and in 10 subjects between 5 and 10 cm. Only one subject had a hair length of 20–30 cm. Hair color was blond for 13 and brown for five subjects.

### 2.2 Imaging Setup and Instrumentation

The first 11 subjects were measured with Elekta Neuromag Vectorview (Elekta Oy, Helsinki, Finland) at a sampling frequency of 600 Hz, and 11 additional subjects were recorded with Neuromag Triux (MEGIN Oy) at a sampling frequency of 1000 Hz. A total of 18 subjects were included in the final analysis, of which nine were measured with the Vectorview machine, and nine were measured with the Triux machine. Both devices comprise 306 channels (204 gradiometers and 102 magnetometers). Figure S2 in the Supplement describes the grand average (18 subjects) MEG power spectral densities (PSDs) averaged over eight channels above the left somatosensory cortex, and the corresponding PSDs for empty‐room measurements from both MEG devices, demonstrating the similarity of the MEG devices' signal‐to‐noise characteristics. The default recording passbands defined by the manufacturer for the applied sampling frequencies were 0.1–172.2 Hz (Vectorview) and 0.1–330 Hz (Triux). The measurements were conducted in a magnetically shielded room (Euroshield, Eura, Finland) at the HUS BioMag Laboratory.

Structural MRIs were mainly acquired at Aalto Advanced Magnetic Imaging (AMI) Center with a 3 T scanner (MAGNETOM Skyra, Siemens Healthineers). The scan included a three‐plane localizer and a T1‐weighted anatomic image (15 subjects). For the remaining three subjects, the MRI images were acquired with Siemens Verio 3T, Siemens Vision 1.5T, and Siemens Espree 1.5T scanners. The locations of the MEG head position indicator (HPI) coils were determined with a 3D digitizer pen (Fastrak, Polhemus, US) and the location of the fiberoptic measurement probe was determined with an in‐house two‐camera photogrammetry system. The head geometry was reconstructed by combining Polhemus and photogrammetry data.

The 16‐channel (16 sources and 16 detectors) frequency‐domain (FD) DOT system developed at Aalto University (formerly Helsinki University of Technology; (Nissilä et al. 2002, 2005)) was used in the DOT measurements. A flexible, non‐magnetic fiberoptic probe was constructed for the simultaneous DOT and MEG recordings, as described in the following. 758 and 824 nm laser diodes were used with a mean optical power of 0.3 mW projected to the tissue surface, and measured amplitude data was used in the analysis.

### 2.3 Fiberoptic Probe Construction

**FIGURE 1:**
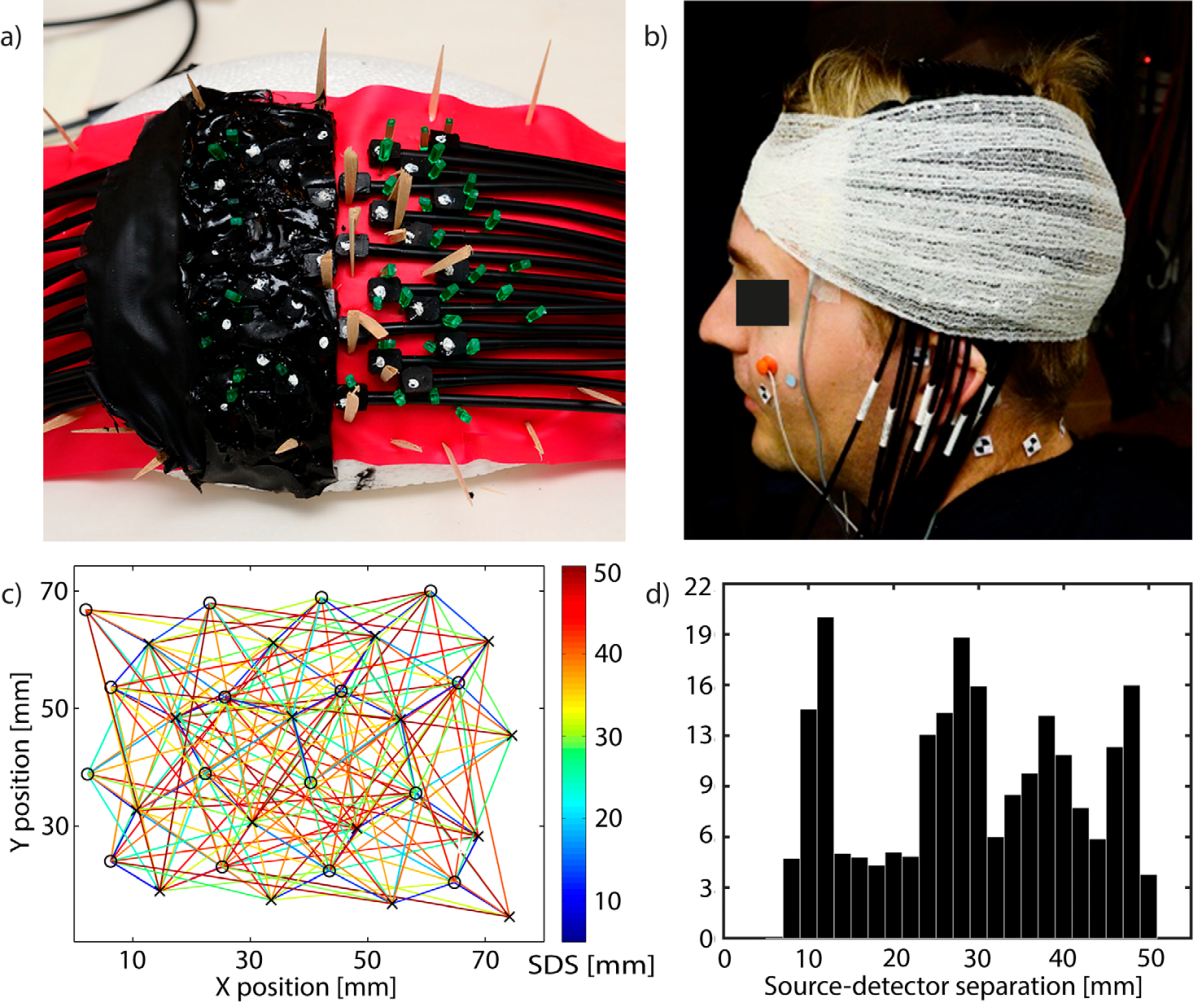
(a) Construction of the high‐density non‐magnetic fiberoptic diffuse optical tomography (DOT) probe. (b) Subject with photogrammetry markers and DOT probe attached. (c) Geometry of the optode arrangement (“X” = source, “O” = detector). Active source‐detector pairs are shown with intersecting lines where color indicates the source‐detector separation (SDS). (d) Histogram of SDSs used for the DOT image reconstruction.

The fiberoptic probe was constructed using custom‐made non‐magnetic optical fiber cables (CeramOptek GmbH, Germany), 3 mm right‐angle prisms (Edmund Scientific) with the black hypothenuse paint chemically removed, and a support material made by mixing carbon black dye powder with transparent silicone (Accutrans Transparent, Ultronics/Coltène). The absorption coefficient of the resulting black silicone was estimated based on transmission measurements with samples of different thicknesses as 17 mm^−1^, which is much greater than tissue absorption (~0.02 mm^−1^) and thus light leaks through the support material are likely to be negligible. The optical leakage rate of the supporting material for the shortest source–detector distances is $∼10^{-20}$. Silicone was cast into molds to make optode terminals, and the material was allowed to cure before removal from the molds. The mold was designed to achieve a low (5 mm) probe thickness to fit inside the MEG helmet and minimize the distance from the scalp to the helmet. The optical fibers, fiber bundles and prisms were inserted into the optode terminals and mounted on a curved styrox surface mimicing the shape of the human head using wooden and plastic toothpicks bent into a V shape to hold the fibers in place, as shown in Figure 1a. After all the fibers and terminals were assembled to the desired geometry, silicone was poured on top of them to fill the gaps, pressed down with a non‐magnetic rubber band, and the silicone was allowed to cure. Finally, the probe was removed from the supports, and the remaining gaps and holes were filled with silicone from both sides. Excess silicone was cut from the edges to finalize the shape of the probe using a single‐use surgical knife; otherwise, only non‐magnetic tools were applied during the manufacturing process. Additional photogrammetry markers were made using Accutrans White (Ultronics/Coltène) on the edges of the top side of the probe. The geometry of source and detector optodes on a flat plane is illustrated in Figure 1c and a histogram of the Euclidean distances between the source and detector positions is depicted in Figure 1d. The routing of the optical fiber cables is shown in Figure S1a and the cross‐sections of the optode terminals are shown in Figure S1b,c in the Supplement.

The finished probe was tested for MEG compatibility by moving it back and forth at a speed of ~2 cm/s near the inside surface of the dewar. Slight disturbances matching the frequency of the probe movement (0.5–1 Hz) were observed over few MEG channels: Since the MEG features of interest correspond to higher signal frequencies and were obtained by averaging over several trials, we did not observe DOT probe‐related artifacts in our MEG signals. The silicone is quite flexible and the probe can be attached to different sizes of heads without difficulty. The probe was designed to support the optical components and minimize signal fluctuation in a situation where slight pressure may be applied to the probe in the radial direction due to contact with the dewar.

### 2.4 Procedure

Prior to the measurement, the five HPI coils were attached to predefined anatomical locations, and marker stickers were placed on the face and at three anatomical landmarks: the nasion and the left and right helix‐tragus junctions. Electro‐oculography (EOG) electrodes were attached for eye movement detection and artifact removal. The approximate head shape, coil and sticker locations, and facial landmarks were registered with the 3D digitizer, and the head was photographed with a stereo camera setup from at least five different orientations. The DOT measurement probe was then attached over the subject's left hemisphere on top of the estimated location of the somatosensory area using a self‐adhesive bandage, and the head with the probe and marker stickers was photographed again (see Figure 1b). The photogrammetry stickers and landmarks were used in post‐processing with the 3D digitizer data to register the DOT measurement probe to the subject's head. The subject was placed inside the MEG device in the supine position with dimmed ambient lighting in the magnetically shielded room. The location of the head with respect to the MEG sensors was determined by feeding current into the HPI coils immediately before each measurement run.

### 2.5 Stimulation

The right median nerve was stimulated with an electrical stimulator (Digitimer DS7A, Digitimer Ltd) near the wrist. The stimulus current amplitude was adjusted to a comfortable level between 7 and 13 mA so that there was a slight but detectable twitch of the thumb. The duration of the stimulus current pulse was 0.2 ms. During stimulation, subjects were instructed to stay still and relax. The stimuli were presented in trains of 2.0002 s duration (measured from the onset of the first stimulus pulse to the offset of the last stimulus pulse of each train), consisting of 2, 3, 5, or 9 stimuli within each train, with inter‐stimulus‐onset intervals of 2, 1, 0.5, and 0.25 s, and corresponding to stimulation frequencies of 0.5, 1, 2, and 4 Hz.

The stimulation frequencies were chosen so that the evoked MEG responses would only minimally overlap with each other even at the highest frequencies, to make identification of different MEG features easier and more reliable. Each stimulus block was followed by a rest period of varying duration (14–65 s) to allow the hemodynamic response to return to the baseline, to reduce the likelihood of phase synchronization with the very low‐frequency oscillations in the background physiology, and to minimize general response habituation. The four different ~2‐s long stimulus blocks were presented in a pseudorandom order within each measurement run so that each stimulus block was presented a total of 15 times within the run, resulting in altogether 60 stimulus blocks per run. Each subject participated in two runs of approximately 25 min, with a resting period of ~10 min between the runs, during which the subjects stayed in supine position under the MEG device.

### 2.6 MEG Signal Processing

The MEG data preprocessing and signal averaging were conducted with the MNE Python software (https://mne.tools/stable/index.html; (Gramfort et al. 2014)). For 15 subjects, the MEG data from two runs was successfully preprocessed, but for three subjects, we were able to analyze only one run of MEG data. In the first subject, one of the MEG runs was rejected due to excessive noise. In two other subjects, one of the two measured MEG raw files was corrupted, and no data from these runs was available.

Artifacts produced by the electrical stimulator were present for about 10 ms after stimulus onset, and they were removed using a median filter. The 50‐Hz power line frequency and its harmonics were removed with a notch filter. The data was then low‐pass filtered at 250 Hz. The filtered MEG data was visually inspected to remove possible noisy periods of data. Independent component analysis (ICA) was used to remove eye‐movement and heart‐related artifacts. The EOG data was typically used to identify via correlation the removable ICA components. For some subjects, the EOG data were missing, and the gradiometer channel data near the eyes were used to identify the waveforms related to the eye blinks. Finally, the signal space separation (SSS) method as implemented in the MaxFilter software (MEGIN Oy, Espoo, Finland) was used to suppress external sources of noise. In subjects with two successful runs, MaxFilter was also used to rotate the head frames of the two runs to the same coordinate frame.

Stimulation‐frequency specific averages were calculated over repetitions of the ~2 s stimulus blocks, starting from 200 ms before the first stimulus, and ending 250 ms after the last stimulus in the train. At the highest stimulation frequency (4 Hz), the signal did not completely return to baseline prior to the onset of the next stimulus. We removed the baseline offset by fitting a piecewise linear function to pre‐stimulus windows from −10 to 0 ms before each stimulus onset, low‐pass filtering the constructed function with a −3 dB cutoff frequency at 8 Hz, and by subtracting it from the averaged time course. The maximum number of averaged stimulus trains per stimulation frequency was 30 for subjects with two runs and 15 for subjects with one successful run. An all‐average response was calculated by averaging over all stimuli across all stimulation frequencies and both runs within subjects.

### 2.7 MEG Source Localization

Equivalent current dipole (ECD) based source modeling was applied for MEG data: It is particularly well suited for modeling somatosensory responses due to the roughly circular shape of the parietal cortex (Nummenmaa et al. 2013) and the strongly dipolar nature of the primary somatosensory responses. ECDs were searched using the XFit software (MEGIN Oy, Espoo, Finland) for the averaged data. The fit was optimized using the anatomical MRIs. The MR images were segmented with FreeSurfer and a uniform spherical conductor model was individually constructed with MRILab (MEGIN Oy, Espoo, Finland). The ECDs were fitted using subsets of gradiometers, starting from time points of the strongest field strength with the most dipolar field shape. Goodness‐of‐fit (GOF) of the selected dipoles over all channels was > 80%. Typically a 3‐dipole model consisting of a dipole at the left SI and dipoles at the left and right SII was applied to the data. For some subjects additional dipoles were found, especially at the posterior parietal cortex (PPC). If needed, the components with the strongest field strengths were first removed from the data to reveal simultaneous sources with smaller field strengths. We assumed that the same cortical sources were activated at the different stimulation frequencies. We thus used the same ECDs, localized based on the all‐average response with the best signal‐to‐noise‐ratio, in all conditions.

### 2.8 MEG Feature Extraction

**FIGURE 2:**
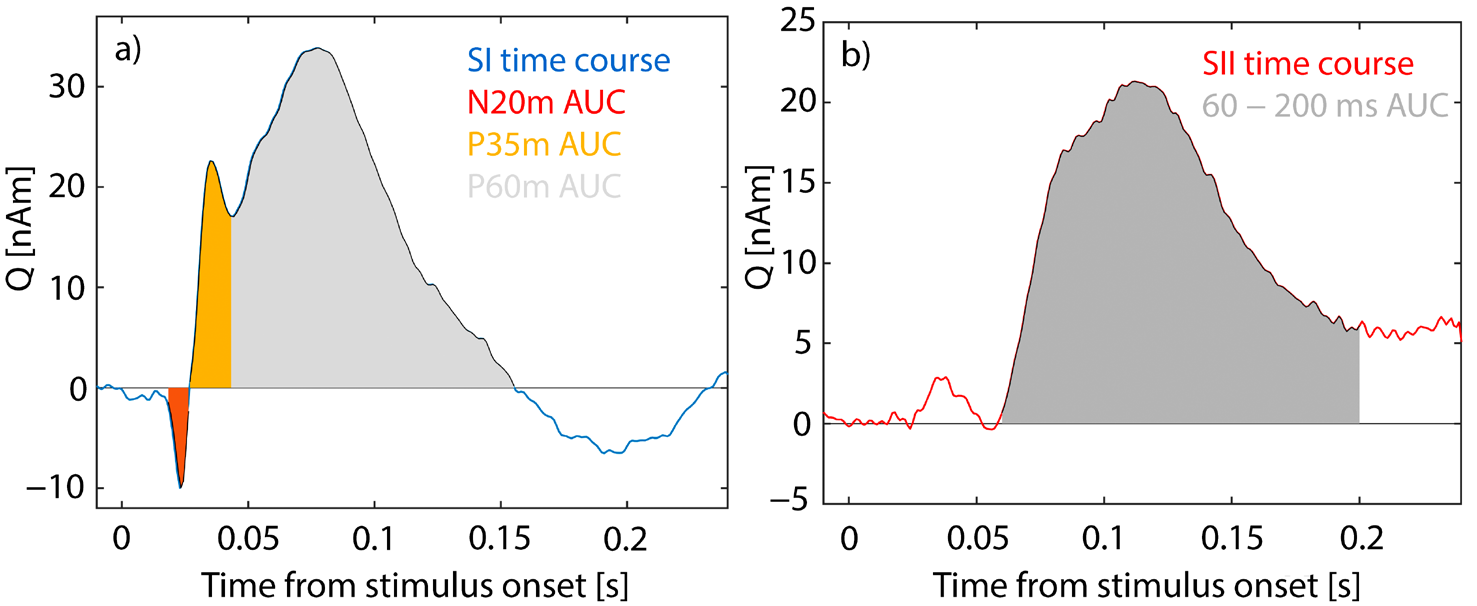
MEG features extracted from the (a) SI and (b) SII ECD time series averaged over all stimulus presentations and subjects.

We calculated the (signed) area under the curve (AUC) for the following features in the dipole time series: For the SI dipole, N20m, P35m, and P60m peaks were individually identified, and the beginning and end time points were determined based on zero crossings or minimums between consecutive signal peaks. For the bilateral SII dipoles, the AUC was determined from the 60 to 200 ms time window. Additionally, root mean square (RMS) values were calculated for the SI and left SII dipoles in the time window of 10–200 ms. The means and standard deviations for the different feature windows are given in Table 1. The SI and SII dipole waveforms averaged over all the subjects and stimuli are depicted in Figure 2.

**TABLE 1:** MEG feature windows. See Figure 2 for an illustration of the ECD waveforms averaged over all stimulation frequencies.

Feature id	Mean beginning point ± SD (ms)	Mean end point ± SD (ms)
SI N20m	18 ± 2	27 ± 2
SI P35m	28 ± 2	40 ± 7
SI P60m	42 ± 8	135 ± 26
SII 60‐200ms	60	200
SI RMS	10	200
SII RMS	10	200

Abbreviation: SD, standard deviation.

### 2.9 DOT Signal Processing

The DOT amplitude signals for each source‐detector pair and wavelength were resampled to a common timebase with a 2 Hz sampling frequency. A natural logarithm was taken of the signal amplitudes and a bandpass filter from 0.01 to 0.33 Hz was applied. The filtered signals were compared with the standard deviation of the signal, and epochs where the absolute value of the filtered log‐amplitude signal exceeded 7 standard deviations were considered artifacts and excluded from the averaging. The FIR deconvolution technique was used to estimate the averaged hemodynamic responses to the four different stimulus train types in a time window from −1 to 10.5 s relative to the stimulus train onset.

### 2.10 DOT Forward Model

Atlas‐based modeling (Heiskala et al. 2007; Custo et al. 2010) is convenient for group‐level analysis, since the reconstructed images can be readily inverse‐transformed back to the common atlas frame. Voxel‐based, individualized head models for DOT image reconstruction were obtained here by registering an individual‐level atlas model to the target points measured with a 3D digitizer and the photogrammetry of the subject's head. The final target point cloud was obtained by solving a combined rotation and translation coordinate transformation that registered the photogrammetry points to matching digitizer points in the least‐squares sense (Autti 2022).

The atlas model was obtained by segmenting the T1 MR images from one of the subjects (male, aged 27) into eight tissue types: scalp, skull, trabecular membranes, subarachnoid cerebrospinal fluid (CSF), CSF in the sulci and ventricles, superior sagittal sinus (SSS), gray matter (GM) and white matter (WM). The segmented model was originally presented in Heiskala et al. (2005), but refined further by separating the sulcus CSF from the subarachnoid CSF to account for the assumed lower density of trabeculae in the sulci with a lower scattering coefficient (Maria et al. 2022; Hirvi, Kuutela, et al. 2023). Manual segmentation was used for all tissue types in the model except the outer boundary of the scalp, which was segmented by thresholding. The atlas landmarks were also picked manually from the MRI. The optical parameters selected for each tissue type are given in Table 2. We used literature values reported for adults at 780–800 nm (Strangman et al. 2003; Heiskala et al. 2005; Custo et al. 2006; Okada and Delpy 2003; Van der Zee et al. 1993). The refractive index was fixed as 1.35, and the anisotropy coefficient was 0.9, if not specified unambiguously in the references.

**TABLE 2:** Tissue‐specific optical parameters used (*Strangman et al. 2003; **Heiskala et al. 2005; ***Custo et al. 2006; ****Okada and Delpy 2003; *****Van der Zee et al. 1993). $\mu_{a}$ is the absorption coefficient, $\mu_{s}$ the scattering coefficient, g the anisotropy factor, and n the index of refraction.

Tissue	$\mu_{a}$ (mm^−1^)	$\mu_{s}$ (mm^−1^)	g	n
Scalp*	0.0164	7.1	0.9	1.35
Skull*	0.0115	9.1	0.9	1.35
Trabecular membranes**	0.016	16	0.95	1.35
CSF (Subarachnoid)***, ****	0.0041	2.5	0.9	1.35
CSF (Sulci and ventricles)****	0.002	0.01	0.9	1.35
Superior sagittal sinus**	0.3	20	0.95	1.35
Gray matter*****	0.032	69.5	0.97	1.35
White matter*****	0.005	55	0.85	1.35

For registering the atlas to the target point cloud for each individual subject, we adapted the process that we have previously implemented for 2‐year‐olds (Hirvi 2019; Maria et al. 2022). We optimized nine registration parameters for the combined scaling, rotation, and translation transformation by minimizing the average of Surface Registration Error (SRE) and Landmark Registration Error (LRE). The error function was modified from Koikkalainen and Lötjönen (2004). Here LRE refers to the average distance between the atlas and target landmarks, with component‐wise thresholds of 3–5 mm to account for the challenge of selecting the exact same anatomical locations from the atlas and the subject. SRE was computed as the weighted average distance from the facial and other head points to the meshed atlas head surface. Less weight (0.8 vs. 1) was put on the facial target points on the cheeks, chin and nose below the nasion, since they reflect facial features more than head shape and are more likely to move even when the head is otherwise still. A triangular head surface mesh (with maximum Delaunay sphere radius of 5 mm) was created with the Iso2Mesh software (Fang and Boas 2009b; version iso2mesh‐1.9.6 downloaded 07/27/2021 from https://github.com/fangq/iso2mesh, which utilizes the Computational Geometry Algorithms Library (CGAL) http://www.cgal.org). We used an iterative closest points approach, thus the closest mesh points were re‐computed after each deformation of the surface mesh instead of minimizing the distance to the originally closest points. The search was performed iteratively in a discrete search space (Mäkelä et al. 2001). The atlas was warped with the obtained parameters, and the locations of the DOT sources and detectors were obtained by interpolation based on the common points of the data sets.

### 2.11 DOT Inverse Problem

The Jacobian matrices for intensity as a function of the absorption coefficients were computed with the “replay” algorithm from the Monte Carlo eXtreme (MCX/MCXLAB) software by simulating $10^{10}$ photon packets for each source in the individualized head models (Fang and Boas 2009a; Yao et al. 2018; v2020 “Furious Fermion” downloaded 08/05/2021 from https://sourceforge.net/projects/mcx/files/mcxlab/). Changes in the voxel‐wise absorption coefficients ($\Delta {\vec{\mu }}_{a}$) were reconstructed from the measured log‐amplitude changes by minimizing the function (Näsi et al. 2013; Heiskala et al. 2009; Maria et al. 2022)

$$\sum_{s,d}{\left(\Delta \log\left[A_{\mathrm{MC}}\left(s,d,\Delta {\vec{\mu }}_{a}\right)\right]-\Delta \log\left[A\left(s,d\right)\right]\right)}^{2}+\alpha {∥\mathrm{L\Delta}{\vec{\mu }}_{a}∥}_{2}^{2}$$

where s and d are the sources and detectors, A the measured amplitude, and $\Delta \log\;\left[A_{\mathrm{MC}}\left(s,d,\Delta {\vec{\mu }}_{a}\right)\right]$ the linear approximation of the amplitude difference based on the Jacobian matrix and the approximation that intensity and amplitude are interchangeable with the chosen modulation frequency of 100 MHz. The term $\alpha ∥\mathrm{L\Delta}{\vec{\mu }}_{a}{∥}_{2}^{2}$ is the Tikhonov regularization term, where the discrete approximation to the negative Laplacian matrix L is (Heiskala et al. 2009)

$$L_{\mathrm{ij}}=\left\{\begin{array}{ll} n & \text{if}\;i=j\\ -1 & \text{if}\;j\;\text{is}\;a\;\text{neighbour of}\;i\\ 0 & \text{otherwise} \end{array}\right.$$

with n being the number of neighboring voxels for i (maximum of 6). Finally, the changes in the absorption coefficients were converted to changes in the concentration of HbO_2_ and HbR according to Cope (1991); The concentration of HbT was calculated by adding the two together.

The DOT field‐of‐view (FOV) was used to limit the voxel‐level statistical testing to a region where we can expect adequate measurement sensitivity. The FOV was defined at the group level to include GM voxels where the mean sensitivity to absorption changes was at least 1% of the maximum sensitivity within the GM.

### 2.12 Clustering

In this work, we present correlation analyses based on voxel‐level HbT responses. To increase the signal‐to‐noise ratio and robustness of our measures, we clustered neighboring voxels based on voxel‐level statistical significance (adapted from Shekhar et al. (2019, 2024); Maria et al. (2020, 2022)). Three different voxel‐wise p value thresholds were used to test the effect of cluster extent. Adjacent voxels with subthreshold p values (thresholds $p_{L1}<0.001$, $p_{L2}<0.003$, and $p_{L3}<0.01$) were merged into clusters. The final correlation coefficient and p value were calculated by averaging voxels in the cluster and choosing the extent and voxel‐level p value threshold (L1, L2, or L3) giving the lowest cluster‐level p value. A minimum cluster size was set at 50 mm^3^ to achieve a false positive rate of approximately 0.05. The cluster‐wise p values were corrected for multiple comparisons with the Bonferroni method with $N_{\mathrm{MC}}=187$ where we estimated the number of regions that can be imaged independently based on the average number of source‐detector pairs used in the DOT reconstructions. The Brodmann areas corresponding to the defined clusters were visually estimated based on Zilles and Palomero‐Gallagher (2020).

### 2.13 Relationship of DOT Response to Median‐Nerve Stimulation Frequency

Motivated by the finding of increased hemoglobin response as a function of increasing stimulation frequency (Tanosaki et al. 2003; Takeuchi et al. 2009), we used MATLAB's corrcoef function to calculate Pearson's correlation between the HbT response AUC and the four stimulation frequencies over all subjects, for each GM voxel within the FOV separately. Neighboring voxels were clustered based on their p values as described in section 2.12 “Clustering”. The locations of possible clusters were also of interest as indirect measures of the success of registering the atlas model to the subject head shape and optode locations.

### 2.14 MEG‐DOT Coregistration

In MEG, the Polhemus landmarks (here nasion and left and right helix‐tragus junctions) define the head coordinate system in which the sensor coil locations are given. The center of the spherical model fitted to the brain MRI and the dipole locations were registered to the MEG head coordinate system using the corresponding landmarks picked manually from the MRI. In DOT, the photogrammetry landmarks define the DOT head coordinate system. The Polhemus and photogrammetry points from each subject's head surface were registered to the atlas frame using the landmarks picked from the atlas MRI. Figure 5a visualizes the SI and SII ECD locations after they were transferred from the MEG head coordinate system to the DOT atlas frame and projected laterally to the atlas brain surface.

### 2.15 Combined MEG‐DOT Correlation Analysis

To find areas where DOT responses share a behavior similar to SI or SII MEG responses, we investigated how voxel‐level hemodynamic responses correlated with the features of the MEG ECD time series. Our approach is analogous to EEG‐informed fMRI analysis where a predicted hemodynamic response is created by convolving selected EEG response waveform features with a canonical hemodynamic response model and associated clusters are located based on the correlation between the measured and predicted hemodynamic signals (Eichele et al. 2005; He and Liu 2008). We focused on HbT as the primary hemodynamic parameter, since it reflects arterioral and capillary diameter changes and is insensitive to metabolism, whereas HbO_2_ and HbR reflect both arteriolar dilation or contraction and changes in oxygen metabolism, resulting in opposite effects on the measured signals. HbT has a greater contribution from the capillary and arteriolar compartments than HbO_2_ or HbR, while HbO_2_ and HbR include greater contributions from the venous compartment than HbT (Hillman et al. 2007). As a result, HbT has been reported to show more focal activation maps than HbO_2_ or HbR in rats (Culver et al. 2005). We considered two different approaches for correlating HbT with the MEG features, referred as Methods 1 and 2 in the following.

In Method 1, the DOT average HbT response, measured as AUC within the time window of 1–9 s after the start of the stimulus block, was placed for each subject and the stimulation frequency in a column vector y for each GM voxel within the FOV as

$$y=\left[\begin{array}{c} {\mathrm{HbT}}_{\mathrm{AUC},\;s1,\;0.5\;\mathrm{Hz}}\\ {\mathrm{HbT}}_{\mathrm{AUC},\;s1,\;1\;\mathrm{Hz}}\\ {\mathrm{HbT}}_{\mathrm{AUC},\;s1,\;2\;\mathrm{Hz}}\\ {\mathrm{HbT}}_{\mathrm{AUC},\;s1,\;4\;\mathrm{Hz}}\\ {\mathrm{HbT}}_{\mathrm{AUC},\;s2,\;0.5\;\mathrm{Hz}}\\ \vdots \\ {\mathrm{HbT}}_{\mathrm{AUC},\;s18,\;4\;\mathrm{Hz}} \end{array}\right]$$

Next we used the MEG feature values, extracted as described in section 2.8 “MEG feature extraction”, to predict hemodynamic responses (AUCs) for comparison with y. The MEG‐predicted time series $m\left(t\right)$ were formed using linear convolution as

$$m{\left(t\right)}_{s,\;f,\;i}=\left(n_{s,\;f,\;i}*h\right)\left(t\right)$$

where t is the discrete time, s the subject identifier, i the MEG feature identifier (see Table 1)

$$\begin{array}{l} i\in \left\{N20m,P35m,P60m,\mathrm{SII}\;60-200\mathrm{ms},\mathrm{SI}\;\mathrm{RMS},\mathrm{SII}\;\mathrm{RMS}\right\} \end{array}$$

$n_{s,\;f,\;i}\left(t\right)$ the time series of the AUC or RMS values extracted for feature i for each stimulus repetition in the block with frequency f, and $h\left(t\right)$ the canonical hemodynamic response function (HRF) model. $n\left(t\right)$ contains a single non‐zero value representing the AUC of the peak for each stimulus in the train for the first four features (the AUCs of the SI waveform peaks around 20, 35, and 60 ms and the AUC for SII in the 60–200 ms window), and for the last two features (the root‐mean‐square (RMS) values for the SI and SII waveforms in the 10–200 ms window), the response waveform is represented by a single non‐zero value equal to the RMS value in $n\left(t\right)$. The HRF was obtained from the Statistical Parameter Mapping (SPM) software package (https://www.fil.ion.ucl.ac.uk/spm/), and adjusted by 0.5 s to obtain a shorter time‐to‐peak than default to compensate for the expected slightly faster HbT response time course compared to BOLD (which is closely related to HbR). $n\left(t\right)$ and $h\left(t\right)$ are formed at a sampling frequency of 20 Hz and resampled to 2 Hz for the calculation of the MEG‐predicted AUC. The predicted HbT waveforms after convolution are shown in Figure 4.

The predicted AUCs based on the linear convolution model using the six MEG features form the columns in the matrix X:

$$X=\left[\begin{array}{ccc} m_{\mathrm{AUC},\;s1,\;0.5\;\mathrm{Hz},\;N20m} & \ldots & m_{\mathrm{AUC},\;s1,\;0.5\;\mathrm{Hz},\;\mathrm{SII}\;\mathrm{RMS}}\\ m_{\mathrm{AUC},\;s1,\;1\;\mathrm{Hz},\;N20m} & \ldots & m_{\mathrm{AUC},\;s1,\;1\;\mathrm{Hz},\;\mathrm{SII}\;\mathrm{RMS}}\\ \vdots & & \\ m_{\mathrm{AUC},\;s18,\;4\;\mathrm{Hz},\;N20m} & \ldots & m_{\mathrm{AUC},\;s18,\;4\;\mathrm{Hz},\;\mathrm{SII}\;\mathrm{RMS}} \end{array}\right]$$

For each voxel, the correlation between the vector y and each of the columns of the matrix $X_{i}$ corresponding to MEG feature i was defined by calculating Pearson's correlation coefficient and corresponding p value with MATLAB's corrcoef function. Adjacent voxels with statistically significant p values were then combined into clusters as described in section 2.12 “Clustering”. Intersubject variability of AUCs was included in the model, so that if a particular subject has a large response to a particular stimulation frequency, and if this behavior is shared between MEG and DOT, it increases the correlation and statistical significance for those voxels.

In Method 2, we considered the similarity of the DOT‐measured and MEG‐predicted hemodynamic response time courses across stimulation frequencies, separately for each of the six MEG features and each individual. Pearson's correlation coefficients were calculated between the DOT‐measured and MEG‐predicted HbT time courses at 20 time points (five time points per stimulation frequency; Figure 4) with a sampling frequency of 0.5 Hz to avoid high temporal correlation of adjacent samples. The correlation coefficients r for each voxel and subject were transformed into approximately normally‐distributed z values using the Fisher transformation

$$z=\frac{1}{2}\;\ln\left(\frac{1+r}{1-r}\right)$$

Student's *t* test was used to evaluate if the transformed voxel‐wise correlation coefficients averaged over subjects had a non‐zero mean. Adjacent voxels with statistically significant correlations according to the voxel‐wise p values were again used to form clusters. Here the overall coupling factor between MEG and DOT responses can differ between subjects, as long as the variability across frequencies is shared.

## 3 Results

All subjects fit comfortably under the MEG dewar wearing the high‐density DOT probe over the somatosensory area, and quality of the DOT and MEG responses obtained were good. With the DOT probe present, the location of the head under the MEG helmet was at most 5 mm lower than in MEG measurements conducted alone, and thus the distance from brain to MEG sensor slightly increased. However, the MEG data quality was good enough for source localization in all 18 subjects. The rejection rate for stimulus blocks in the MEG analysis was 9.8% ± 9.3% (mean ± SD) and in the DOT analysis, 9% ± 6% (mean ± SD).

**FIGURE 3:**
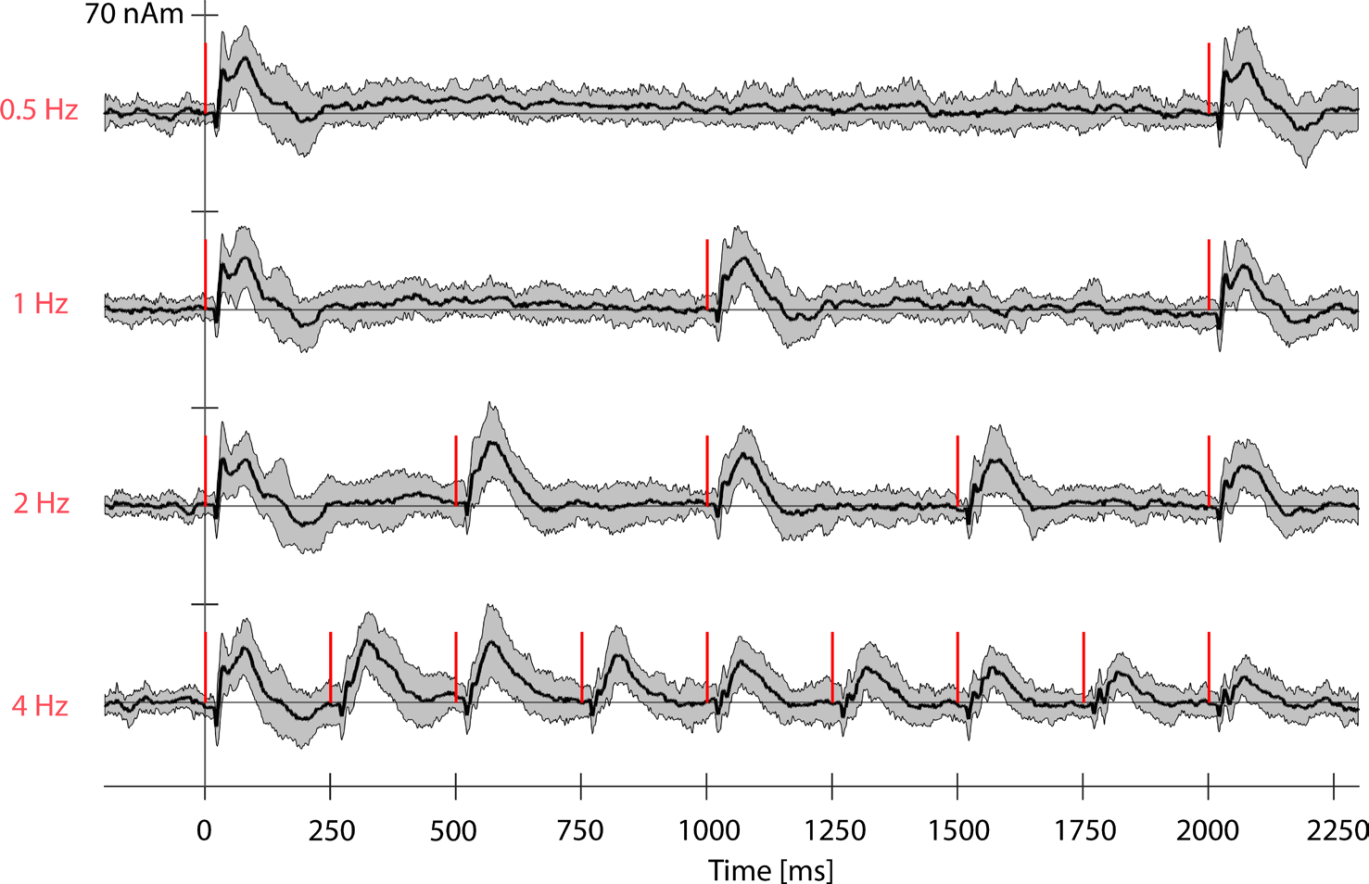
MEG left‐hemispheric SI dipole waveforms averaged over 18 subjects for each stimulation frequency for the duration of the ~2‐s stimulus train. The red bars indicate the individual stimuli given during the stimulus trains and the gray shading shows the standard deviation over subjects.

Figure 3 shows the MEG SI ECD waveforms for all four stimulation frequencies averaged over 18 subjects. The SI and SII ECD locations for individual subjects registered to the atlas frame are shown in Figure 5c–g, and projected to the brain surface in Figure 5a. SI dipoles were typically located around area BA3b and the SII dipoles around BA40/43.

**FIGURE 4:**
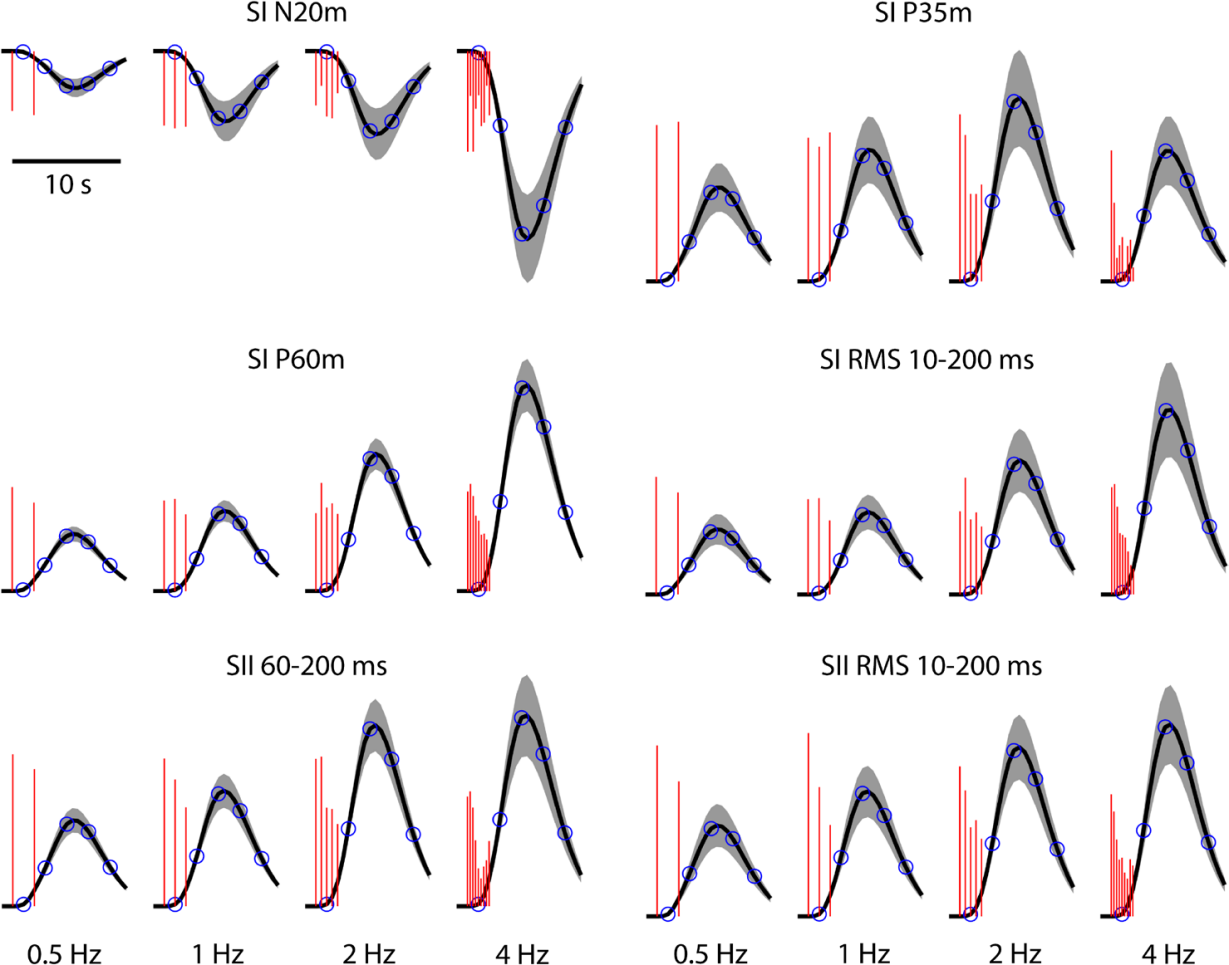
MEG response feature values corresponding to each individual stimuli (illustrated as red vertical lines, height indicates the AUC or RMS value) and the corresponding predicted hemodynamic responses (black). Shading indicates the standard deviation of the responses over subjects, and blue circles indicate the time points at which the predicted hemodynamic response was sampled. AUCs of the hemodynamic responses were calculated within the time window of 1–9 s (Method 1). Five samples were extracted from the hemodynamic responses at 1, 3, 5, 7, and 9 s post stimulus train onset (Method 2).

Figure 4 illustrates the time series of the AUC/RMS values of the MEG features at the four stimulation frequencies, and together with their predicted hemodynamic responses. The resulting curves were used for correlation analysis with Methods 1 and 2. The different habituation characteristics of the MEG features are evident by observing the decay of the red vertical lines as a function of time in Figure 4.

**FIGURE 5:**
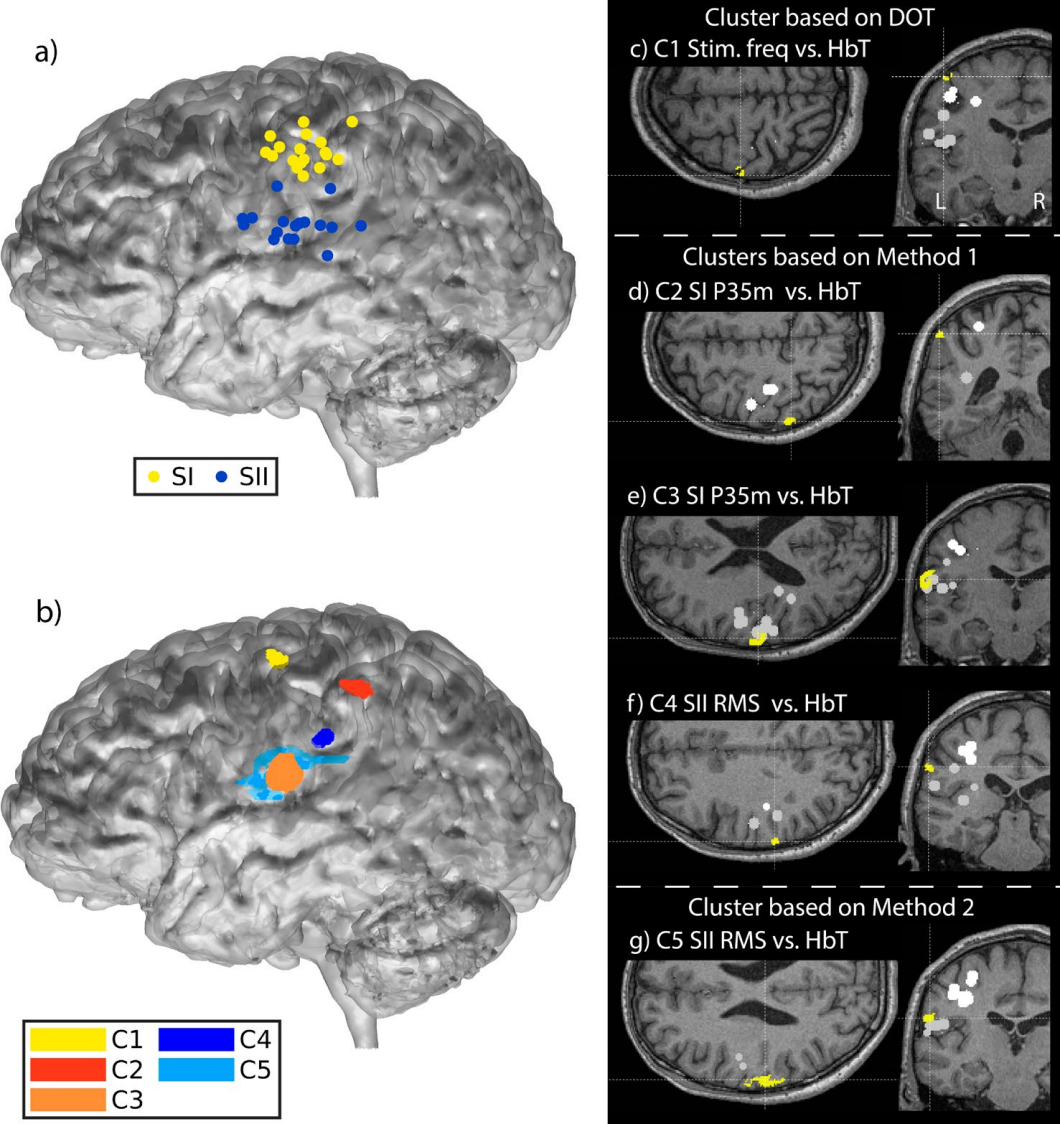
Three‐dimensional rendering of the atlas gray matter surface with (a) MEG ECDs and (b) clusters based on correlation with measured and predicted hemodynamic responses. Axial and coronal slices of the atlas brain with cluster (c) C1 (Brodmann area (BA) 1, BA4) based on correlation between stimulation frequency and HbT area‐under‐the‐curve (AUC), and clusters (d) C2 (BA2, BA7), (e) C3 (BA40, BA43), (f) C4 (BA40), and (g) C5 (BA40, BA43) based on correlation between MEG features and HbT AUC (d–f) or time course (g). The cluster locations in subfigures (c–g) are marked in yellow, SI ECDs with white spheres, and SII ECDs with light gray spheres. See Tables 3 and 4 for detailed information on the clusters.

The clusters that were found based on the correlation between DOT HbT and the stimulation frequency, and the DOT HbT and hemodynamic activity predicted using time‐course characteristics of the MEG sources and the canonical hemodynamic response model, are shown in Figure 5, and Tables 3 and 4. The significance level threshold for reporting the results was set at p < 0.2 (after correcting for multiple comparisons with the Bonferroni method with $N_{\mathrm{MC}}=187$). The clusters with p < 0.05 have been marked with an asterisk (*) in Tables 3 and 4. By correlating the stimulation frequency and HbT response, Cluster 1 was located over the hand area of SI in BA1/BA4 (HbT and stimulation frequency r = 0.38, p = 0.16; HbO_2_ and stimulation frequency r = 0.39, p = 0.14).

**TABLE 3:** Cluster found based on correlation between hemodynamic response and stimulation frequency. Cluster location and size, and Pearson's correlation coefficients (r) and p values for the correlations of HbT, HbO_2_ and HbR response AUCs with stimulation frequency are given. p values are reported after multiple comparison correction. p values ≥ 0.20 are indicated as not significant (NS).

Cluster	Location/Vol.	HbT	${\mathrm{HbO}}_{2}$	HbR
C1 (*N* = 18) (Figure 5b–c)	Parietal BA1, BA4			
	0.06 ${\mathrm{cm}}^{3}$	r = 0.38	r = 0.39	r = −0.22
Leading DOT: HbT	L3	p = 0.16	p = 0.14	NS

**TABLE 4:** Clusters found based on statistically significant correlation between the optically measured hemoglobin response time courses and the time courses predicted based on linear convolution between MEG SI and SII ECD features and the canonical hemodynamic response function (Methods 1 and 2). Clusters C2–C4 were found based on the hemodynamic response area under the curves (AUCs; Method 1), and cluster C5 based on point‐wise correlation over the time course (Method 2). Cluster location and size, Pearson's correlation coefficients (r) and p values for the correlations of the leading MEG feature and HbT, HbO_2_ and HbR responses are given. *N* = 17 for clusters 4 and 5 due to the voxels in these clusters being outside of one subject's FOV. p values have been corrected for multiple comparisons with the Bonferroni method (*N*_MC_ = 187). Statistically significant correlation (p<0.05) is indicated with an asterisk (*). p values ≥ 0.20 are shown as not significant (NS). Voxel‐wise clustering thresholds are indicated with L1 (p< 0.001), L2 (p< 0.003), and L3 (p< 0.01).

Cluster	Location/Vol.	HbT	${\mathrm{HbO}}_{2}$	HbR
C2 (*N* = 18) (Figure 5b,d)	BA2, BA7			
Leading MEG: P35m	0.1 ${\mathrm{cm}}^{3}$	r = 0.44	r = 0.13	r = 0.21
Leading DOT: HbT	L2	p = 0.021*	NS	NS
C3 (*N* = 18) (Figure 5b,e)	BA40, BA43			
Leading MEG: P35m	0.64 ${\mathrm{cm}}^{3}$	r = 0.46	r = 0.37	r = −0.10
Leading DOT: HbT	L1	p = 0.011*	NS	NS
C4 (*N* = 17) (Figure 5b,f)	BA40			
Leading MEG: SII RMS	0.11 ${\mathrm{cm}}^{3}$	r = 0.41	r = 0.42	r = −0.28
Leading DOT: HbT	L2	p = 0.098	p = 0.064	NS
C5 (*N* = 17) (Figures 5b,g and 6)	Parietal BA40, BA43			
Leading MEG: SII RMS	0.84 ${\mathrm{cm}}^{3}$	r = 0.22	r = 0.13	r = 0.0059
Leading DOT: HbT	L3	p = 0.014*	NS	NS

**FIGURE 6:**
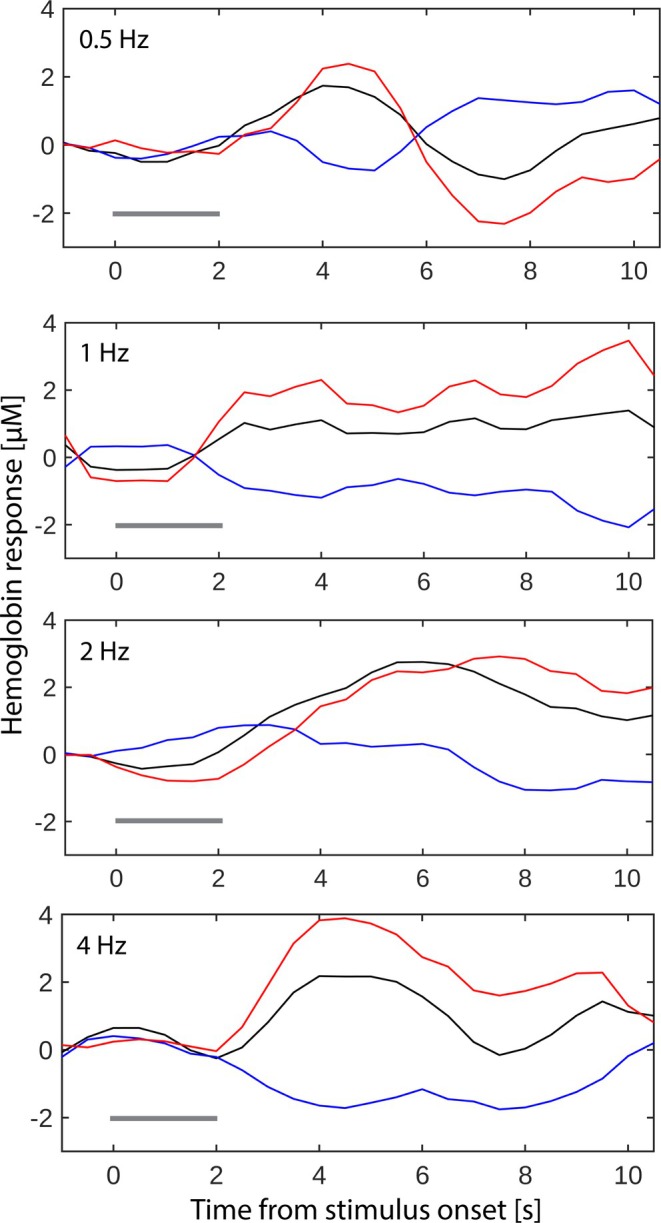
Measured hemodynamic response time courses for cluster C5 with HbT in black line, HbO_2_ in red line and HbR in blue line. The stimulation period 0–2 s is indicated with a dark gray horizontal line. The stimulation frequency is indicated in the top left corner of each plot. (See Table 4; Figure 5b,g).

Table 4 describes the clusters for which the tested MEG features correlate with HbT values, based on correlation analysis with Methods 1 and 2. Clusters 2 and 3 are based on correlation with MEG SI P35m (Figure 5b,d,e) and reside on areas BA2/BA7 and BA40/BA43, respectively. Cluster 4 results from correlation with SII RMS (Figure 5b,f) and resides on area BA40. Cluster 5 also results from correlation with SII RMS (Figure 5b,g) and resides on area BA40/BA43. Figure 6 shows an example of the time courses for HbT, HbO_2_, and HbR, averaged over all voxels in cluster 5. The measured HbT time course corresponding to 4 Hz trains has a stronger transient component than the 1 and 2 Hz trains at around 2–7 s, in line with the stronger and faster decay of MEG SII RMS at 4 Hz (Figure 4). No clusters were found to correlate with SI N20m, SI P60m, SII 60–200 ms or SI RMS features. In addition to HbT, ${\mathrm{HbO}}_{2}$, and HbR correlations within the clusters are given in Table 4.

The correlation coefficients for the statistically significant clusters were recalculated for the two subgroups of subjects measured with the Vectorview and Triux systems and compared with the permutation test to confirm that the correlation coefficients did not differ statistically significantly between the subjects acquired with the two systems.

## 4 Discussion

In this work, we demonstrate the usability of a multimodal imaging technique, where MEG is combined with HD‐DOT to simultaneously record magnetic fields and hemoglobin responses related to somatosensory stimulation. We also describe the construction of a non‐magnetic high‐density fiberoptic probe.

Our results suggest that the information obtained from simultaneously recorded neuronal and hemodynamic responses can be informatively coupled. We brought the data from the two functional imaging modalities together and investigated which areas within the field of view of the DOT imaging probe exhibited significant correlations between HbT changes and MEG features. We assumed the relationship to be approximately linear at the source level, and macroscopic nonlinearities between the modalities to arise mainly from modality‐dependent spatial sensitivities and the presence of both inhibitory and excitatory connections. We searched for clusters of gray matter voxels where the HbT responses correlate with MEG SI and SII ECD features across subjects and stimulus conditions, or across stimulus conditions within each subject. We found two clusters near the postcentral gyrus based on the correlation between the measured HbT AUC and the MEG‐predicted HRF AUCs calculated from the SI 35 ms MEG responses, and a cluster in the inferior edge of the postcentral gyrus and parietal operculum based on the correlation between HbT and the MEG SII RMS value. All the statistically significant clusters were close to the postcentral gyrus, which is the expected location of the somatosensory cortex. Importantly, the constructed DOT probe did not cause any significant artifacts in the MEG responses: The observed location of SEFs and their behavior to median nerve stimulation at various frequencies was in good accordance with earlier somatosensory MEG studies (e.g., Mauguiere et al. (1997); Raij et al. (2003); Wikström et al. (1996)). Furthermore, the high stimulus acceptance rates of DOT responses indicate that the probe supported the optical fibers well and effectively reduced motion artifacts due to mechanical contact with the MEG helmet.

The clusters showing correlating activity between the imaging modalities are likely to point to the original sources of the MEG activity and/or to regions connected with them. Although hemodynamic responses are known to be modulated by neuronal activity, the complex relationship between hemodynamic and neural activity and also the attenuation of hemodynamic responses to frequent stimulation is not yet fully understood. We searched for voxels where the HbT response correlates with the frequency of stimulation and found a cluster with suggestive positive correlation over the hand area in the (post)central gyrus. In the current implementation of DOT based on amplitude data, the center of gravity of deep activations moves toward the surface of the cortex and the reconstructed contrast is reduced due to the characteristics of the method. Since MEG is most sensitive to tangential current sources in the sulci, possible differences in DOT and MEG responses in the same apparent source region might not always indicate actual nonlinearities in the neurovascular coupling; instead, such effects could arise from the different spatial sensitivity patterns of the two methods.

The lack of hemodynamic responses with significant correlation with the earliest MEG response around 20 ms after stimulus onset (N20m) is consistent with previous findings by Ou et al. (2009) who noted that the hemodynamic response attenuated more strongly to repeated stimulation than the N20m response, while later MEG responses provided a better correlation with the hemodynamic response. We suggest two additional explanations for this. On the one hand, the cortical neurons that activate first at around 20 ms reside fairly deep in the sulcus where DOT has low sensitivity. On the other hand, the 20 ms response may reflect high level of synchrony in the initially activated neuronal population, while the hemodynamic response may be more related to overall synaptic activity with less sensitivity to temporal synchrony. However, using higher stimulation frequencies (10 Hz) than applied here, Takeuchi et al. (2009) reported EEG current source estimates at 22 ms to coincide with the NIRS HbO_2_ response at the contralateral SI hand area.

The hemodynamic responses predicted on the basis of MEG SII activity were located around BA 40/43, that is, secondary somatosensory cortex. The highest correlations between HbT and MEG features were found using the SI P35m peak and BA 40/43. This may suggest that the neurons giving rise to the P35m peak in SI could have axons targeting the inferior end of the postcentral gyrus and/or the SII cortex.

Most of the DOT responses that correlated with MEG features were located in the inferior part of the postcentral gyrus close to the region where MEG SII activity was located. Huppert et al. (2017) made spatial comparisons between fMRI BOLD responses, cortically constrained reconstructions of HbO_2_ and HbR, and MEG source activity to median nerve stimulation, and reported average displacements in the centers‐of‐gravity between reconstructed optical and MEG activations to be 27 mm for HbO_2_ and 29 mm for HbR, while the displacements were somewhat smaller between HbR, HbO_2_ and fMRI BOLD. The regions of interest for fNIRS HbO_2_ and HbR were somewhat inferior to the MEG activation. The authors also reported additional fNIRS activation in frontal areas, measured by HbO_2_ and HbR. While we concentrated here on HbT measures as they are more closely confined to areas with neuronal activity (Culver et al. 2005; Hillman et al. 2007), based on correlation between HbO_2_ (and HbR) versus MEG SI P35m, we also found a cluster more frontally near the premotor cortex. However, because of an adjacent large vessel and the absence of statistically significant correlation of the area with HbT which was our primary hemodynamic parameter of interest, we did not include these in the results.

We recommend to use HbT as the DOT metric of brain activity rather than HbO_2_ or HbR: in our experience, the HbT results in reconstructed images tend to be more precise in location and easier to interpret. Ideally HbT, which is closely related to CBV, would be paired with relative cerebral metabolic rate of oxygen (rCMRO_2_) as these two parameters represent the vascular and metabolic responses rather than mixed signals like HbO_2_ and HbR (Roche‐Labarbe et al. 2014).

### 4.1 Limitations and Future Improvements

Given the desired high optode density to achieve good image quality in the region studied and the available 16‐channel instrument, we are currently limited to imaging a part of the cortex that constitutes ~10%–20% of the total gray matter volume in adult subjects. Also the imaging depth of DOT is relatively shallow for adult subjects, but some sensitivity is expected deeper in the sulci due to the low‐scattering CSF. Accurate characterization of sensitivity in the sulci is subject to further research. The optical power of the light sources was lower than ideal for adult human subjects, and future studies should be made with a more optimized configuration with increased source power and greater numerical aperture of the detector fiber bundles. The use of phase data in addition to the amplitude measurements can improve spatial accuracy and depth resolution provided that the detected optical power is adequate (Hirvi, Nissilä, et al. 2023). The FOV of the imaging would ideally be defined using criteria based on reconstruction accuracy instead of measurement sensitivity. Increased optode density and FOV can be achieved in the future via the development of new instrumentation. While this has the potential of improving the image quality (Heiskala et al. 2009), a greater number of optical fibers and bundles may make the measurements less comfortable for the subjects.

Thick, curly hair may cause challenges with our current probe due to poor contact with the scalp and air gaps leading to light leaks, reducing the contrast‐to‐noise ratio for brain signals. A possible solution might be to use time‐domain technology and leave out the photons with the shortest propagation times from the analysis. A larger study population would be needed to comprehensively analyze the impact of hair color, type, and length on the DOT stimulus rejection rate using the presented MEG‐compatible probe.

Automated segmentation of extracerebral tissues is a topic of future development in DOT head modeling. It would potentially enable the use of each subject's own MRI for more accurate forward modeling, which would also benefit from more accurate estimates for the tissue‐specific baseline optical parameters. However, the atlas model provides a more straightforward framework for group‐level analysis of responses in gray matter than individual MRIs. Custo et al. (2010) observed that the responses to median nerve stimulation were reconstructed at the same gyri based on the individual MRIs and the linearly registered atlas model. In the current work, the MEG‐DOT clusters aligned roughly with the postcentral gyrus in the atlas model.

Higher stimulation frequencies and longer stimulus trains than used in the present study could produce larger hemodynamic responses. However, this would lead to greater overlap of MEG responses at higher frequencies, making the extraction of features more difficult.

Although a linear relationship between MEG and DOT evoked response features across conditions was used as the basis of the present analysis, a perfectly linear relationship is not necessary for the approach. A greater number of stimulus block repetitions, along with an increase in SNR, would be needed to precisely characterize the linearity of the relationship between MEG and DOT responses. To achieve this, further improvements in the instrumentation and optimization of the study protocol are required.

The voxel‐based clustering approach favors focal activations as it starts from high significance in individual voxels and combines adjacent voxels into clusters. The earliest cortical activity caused by median nerve stimulation can be detected at around 20 ms post stimulus onset, and it then spreads to the surrounding areas, leading to progressively more distributed activity at later time points. Thus, the voxel‐based clustering algorithm emphasizing focal activations may highlight the early MEG features such as the P35m. A different approach searching for correlations starting from larger regions such as the whole field‐of‐view of the DOT probe, anatomically defined regions of interest such as the postcentral gyrus, and continuing toward smaller regions might lead to additional correlations related to the distributed activity at later time points during the spatiotemporal activation sequence. As a complement to the time‐domain features calculated here from the evoked responses, other approaches such as time‐frequency analyses may allow the identification of additional features of interest for MEG‐DOT analysis.

We believe that MEG‐DOT in general and the approach presented here may have future applications, for example, to locate the origins of electrophysiological signals and as an alternative technique for imaging functional connections. In addition to the evoked responses investigated in the present study, MEG‐DOT may be applicable to the study of functional connections, for example, via correlating oscillatory activity within and across modalities.

Compared to EEG‐DOT, MEG‐HD‐DOT provides a high‐density of optodes and the magnetic field sensors over the same area of the cortex without difficulty. Also, the experimental preparations are easier and faster on the subject, for example, no scratching of the scalp is necessary to achieve low impedance coupling as with EEG electrodes. While MEG has a higher spatial resolution than EEG, the latter may more easily see signals from the gyri. EEG systems are also much more widely available and less expensive than MEG systems.

In the future, a novel MEG technology based on optically pumped magnetometers (OPMs) may be used in multimodal studies (Ru et al. 2022). OPMs have already been used to measure magnetic fields evoked by median nerve stimulation (Iivanainen et al. 2019). Ru et al. (2022) were the first to validate the individual performance of OPM‐MEG, EEG, and fNIRS in simultaneous measurements with their own in‐house instrumentation in a visual checkerboard experiment with five healthy adults. OPM helmets can be more easily adopted to different head sizes. Placing the DOT probe between the scalp and the OPM sensors would effectively insulate the subject from the sometimes problematic heat generated by OPMs, but the signal would be reduced due to the increased distance from the brain. Alternatively, interlacing the OPMs and optodes on the scalp may be possible but would likely reduce the density of the optode arrangement. Finally, wearable applications for DOT are of increasing interest (Vidal‐ Rosas et al. 2023), and wearable OPM MEG helmets have also been implemented (Seymour et al. 2021; Brookes et al. 2022). Hybrid OPM‐MEG, EEG, and DOT in a wearable helmet may provide a promising future step.

## 5 Conclusion

We showed that high‐density diffuse optical tomography and magnetoencephalography measurements can be performed simultaneously, here demonstrated by recording responses to electrical median nerve stimulation. We also suggested ways to correlate the MEG and DOT activations. Our results show that the SI P35m MEG response correlates with the total hemoglobin response over the SII region and an area close to the hand “knob” of the SI. Furthermore, the hemodynamic response time series predicted from the MEG SII activity correlated with HbT in Broadmann areas 40/43 or the SII. Limitations and future directions of the methodology were also discussed.

We suggest that this multimodal technology may be used to identify anatomical locations that are possible sources of specific features of the MEG/EEG electromagnetic signals and to understand the coupling between electrophysiological and hemodynamic signals. We think the technique has potential, especially for studies in children.

## Ethics Statement

The study was approved by the Helsinki University Hospital Regional Committee on Medical Research Ethics. Written informed consent was obtained from each subject prior to measurement.

## Conflicts of Interest

The authors declare no conflicts of interest.

## Supporting information


Data S1.

## Data Availability

The brain imaging data cannot be made publicly available due to ethical restrictions imposed by the research ethics committee statement and Finnish law. Relevant derived and pseudonymized data supporting the findings can be shared upon reasonable request and with a permission from the research ethics committee for research aiming to reproduce the results. Custom code used in the analysis is available from the corresponding author upon reasonable request.
