# Supplementary material for: Simultaneously Acquired Magnetoencephalography and Diffuse Optical Tomography Data Reveals Correlated Somatosensory Activity
Source: Hum Brain Mapp. 2025 Jul 27;46(11):e70293. doi: 10.1002/hbm.70293 (PMC12301506; doi:10.1002/hbm.70293)
Supplement: Supplementary file 1 — Data S1. [file HBM-46-e70293-s001.pdf]

Supplement to Simultaneously acquired magnetoencephalography and diffuse optical tomography data reveals correlated somatosensory activity

Salla Autti[a,b], Pauliina Hirvi[c], Mariia Keitaanniemi[a], Hanna Mustaniemi[a], Kalle Kotilahti[a], Hanna Renvall[a,b], Ilkka Nissilä[a,*]

[a] Aalto University, Department of Neuroscience and Biomedical Engineering, Finland

[b] BioMag Laboratory, HUS Medical Imaging Center, Aalto University, University of Helsinki and Helsinki University Hospital, Finland

[c] Aalto University, Department of Mathematics and Systems Analysis, Finland

* Corresponding author: Ilkka Nissilä, Aalto University, Department of Neuroscience and Biomedical Engineering, P. O. Box 12200, FI-00076 AALTO, Finland, ilkka.nissila@aalto.fi

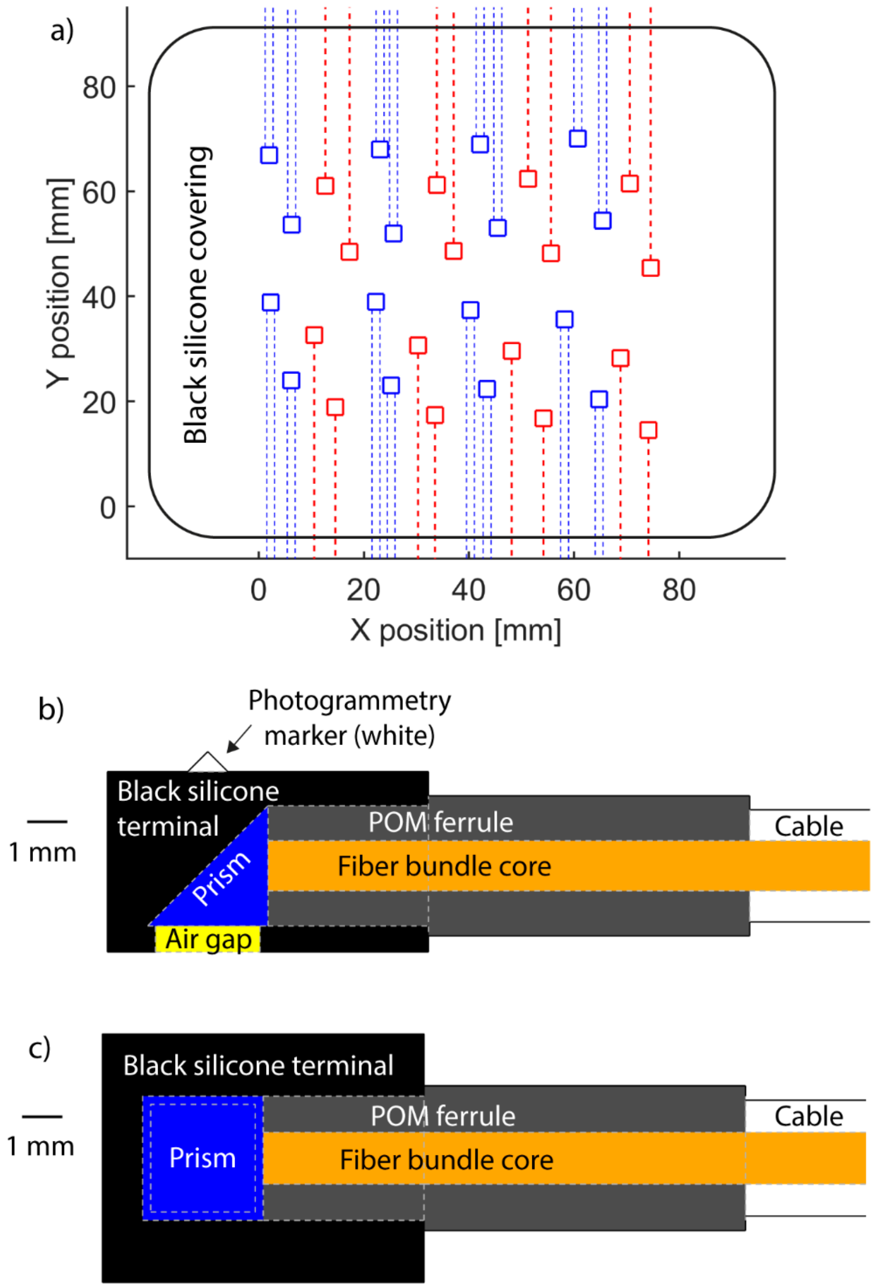

Figure S1: a) Layout of the source (red square) and detector (blue square) prisms and optical fiber routing across a cross-section of the non-magnetic, high-density, fiberoptic diffuse optical tomography probe. b) Cross-section of a silicone prism terminal (black) with prisms (blue), fiberoptic bundle terminals made of black polyoxymethylene (POM; dark gray), the optical fiber bundle core (orange), and the air gap between tissue and prism (yellow; side view). c) Orthogonal cross-section of the prism terminal shown from the top.

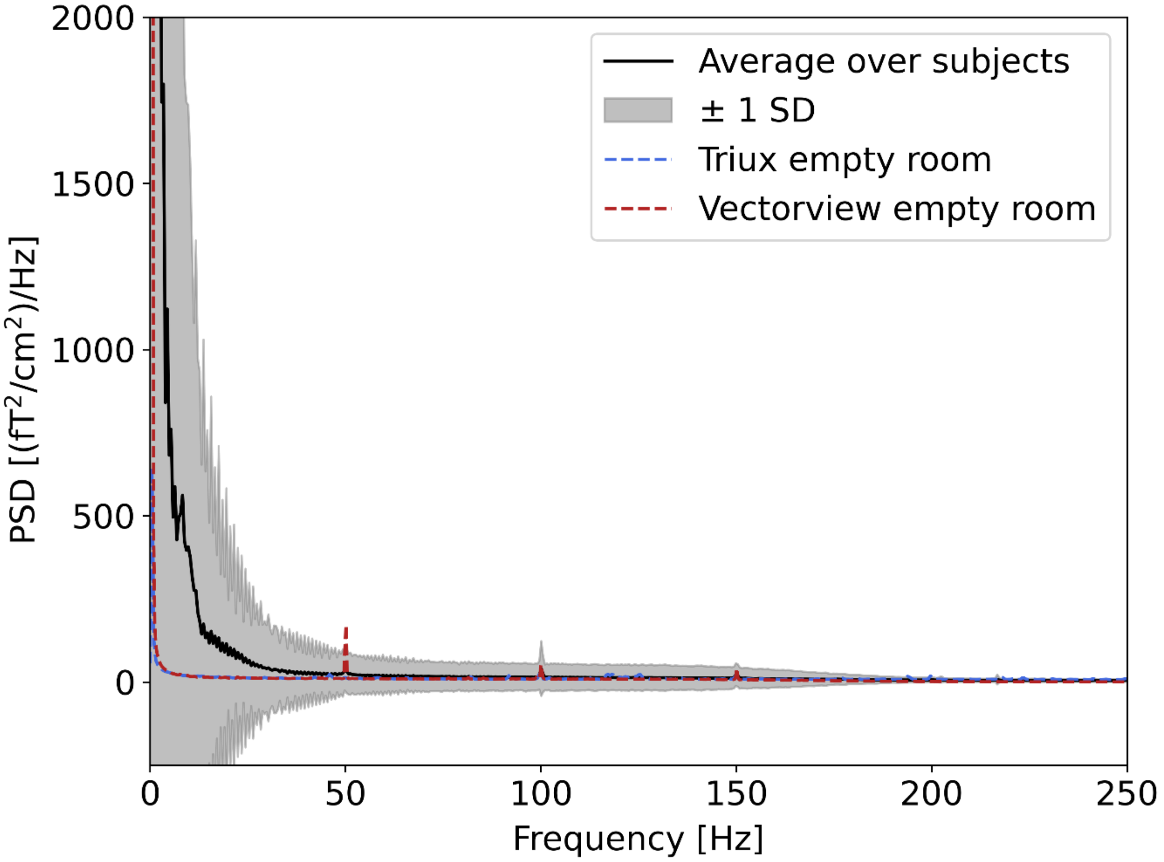

Figure S2. Mean power spectral density (PSD) of raw data averaged over 18 subjects and eight gradiometer channels residing over the left somatosensory cortex are shown with the black curve (± standard deviation is indicated with gray shading). PSDs based on empty-room recordings over the same channel selection for the Triux (blue dashed) and the Vectorview (red dashed) devices are also depicted. Please note that the presented data has not been preprocessed and thus includes the 50-Hz line frequency and its harmonics.
